# Paratuberculosis: a mucosal immunology perspective

**DOI:** 10.3389/fimmu.2026.1845415

**Published:** 2026-06-30

**Authors:** Itzel Aguilar-Lopez, Laura Gomez, Antonio Facciuolo

**Affiliations:** 1 Vaccine & Infectious Disease Organization (VIDO), University of Saskatchewan, Saskatoon, SK, Canada; 2 Department of Veterinary Microbiology, Western College of Veterinary Medicine, University of Saskatchewan, Saskatoon, SK, Canada

**Keywords:** Johne’s disease, mucosal immunity, paratuberculosis, Peyer’s patches, vaccine

## Abstract

Johne’s disease, caused by *Mycobacterium avium* subsp. *paratuberculosis* (MAP), is a chronic enteric infection of ruminants characterized by persistence in intestinal macrophages following invasion through Peyer’s patches. Early studies established a predominantly cell-mediated immune response during early stages of infection, yet this fails to clear mucosal infection. Recent vaccine studies demonstrate partial, region-specific protection and induction of mucosal immune responses but also reveal marked differences in responses across intestinal regions. Correlates of protection are poorly defined and key knowledge gaps remain in defining the mechanisms underlying mucosal immunity to MAP in ruminants. In this mini review, we synthesize current knowledge of host immune responses to MAP and highlight research gaps from a mucosal immunology perspective that continue to impede vaccine development. Addressing these gaps is essential to advance vaccine research for Johne’s disease.

## 1 Etiology, epidemiology, and impact of MAP infection

*Mycobacterium avium* subsp. *paratuberculosis* (MAP) is a bacterial pathogen that establishes persistent, incurable intestinal infections in ruminants (1) for which no effective treatment or vaccine exists (2). Infection usually occurs early in life through ingestion of MAP contaminated milk, feed, or water (3). Infected animals may remain asymptomatic for years, intermittently shedding MAP in feces or in milk (4). Disease progression can lead to Johne’s disease (JD), a chronic granulomatous gastroenteritis characterized by diarrhea, weight loss, and eventual death. Many infected animals remain undetected due to limited diagnostic sensitivity (5). Despite the absence of clinical signs, infection negatively impacts productivity. Infected cattle produce less milk (6), have reduced reproductive efficiency, lower slaughter value, and are more likely to be prematurely culled leading to economic losses (7, 8). Food safety concerns and zoonotic risk have been raised because of MAP resilience in soil (9) and water, resistance to water treatment processes (10), and survival through pasteurization (11).

Global economic losses from JD are estimated at over USD 4 billion annually (12), though likely an underestimate due to diagnostic tests with variable sensitivity (13, 14). Herd-level prevalence varies across regions, exceeding 40% in dairy cattle, generally lower in beef cattle (5), and over 50% in sheep (15). MAP persistence in intestinal tissues requires an understanding of mucosal immune responses governing these interactions. This mini review summarizes current knowledge of innate and adaptive immunity to MAP, emphasizing mucosal responses, and highlights major research gaps, viewed through the lens of mucosal immunity, that must be addressed to advance vaccine development.

## 2 Mucosal immunity of paratuberculosis

### 2.1 Host-MAP interactions: innate immunity

Following ingestion, MAP breaches the intestinal barrier via microfold cells (M cells) located in Peyer’s patches (PP) (16) occurring as early as 30 minutes post-exposure in calves (17) and goats (18). After transcytosis, MAP is phagocytosed by subepithelial phagocytes and can rapidly disseminate to mesenteric lymph nodes (LN) within one-hour (19). M cell interaction is mediated by fibronectin bridges linking integrins with MAP fibronectin-attachment proteins (20). Fibronectin-independent attachment is mediated by mammalian cell entry proteins (21), glyceraldehyde 3-phosphate dehydrogenase protein (22), and oxidoreductase proteins (23). MAP can invade through enterocytes as shown with goat ligated intestinal segments (24, 25) and supported by *in vitro* models including bovine epithelial cells (26) and intestinal organoids (27, 28). Epithelial cell passage can enhance MAP survival and uptake in macrophage (29).

MAP survives and replicates within macrophages by modulating intracellular pathways to evade immunity (30). After phagocytosis, MAP blocks phagosome maturation (30) by maintaining early endosomal markers and reducing acidification (31). MAP suppresses pro-inflammatory responses, including IFN-G (32) and NF-kB signaling (33), to limit macrophage activation while impairing CD40 signaling (34) which is essential for macrophage-T cell interactions. MAP promotes anti-inflammatory IL-10 (35), impairs antigen presentation via downregulation of major histocompatibility complex (MHC) class II, and inhibits apoptosis through caspase signaling pathways (36, 37). Infected bovine monocyte-derived dendritic cells (DC) exhibit a semi-mature phenotype with impaired endocytic function, reduced MHC class II antigen presentation, and low T cell activation (38). Studies of MAP using mucosal macrophages and DCs are limited, innate immune modulation has primarily been characterized in blood-derived mononuclear phagocytes.

In calf intestinal loop models, MAP reduces expression of mannose and complement phagocytosis receptors, decreases phagosome-lysosome fusion, and skews responses towards T helper 2 (Th2) cells (39). In calves, MAP downregulates MHC class II antigen presentation in systemic leukocytes, while upregulating MHC class I (37) Monocytes and monocyte-derived macrophages (MDM) from JD-positive cows display a tolerance-like phenotype (40) with increased IL-10 response to MAP antigen (41).

γδ T cells are early responders during MAP infection (42). High levels of γδ T cells are observed in blood (43) and increased γδ T cells are found in the intestinal lamina propria, specifically WC1^+^ subsets within the epithelial layer (44). Focal lesions, versus diffuse lesions, contain more WC1^+^ subsets suggesting an association with better control of infection and disease progression (45). WC1^+^ subsets co-cultured with MAP-infected MDMs enhance nitrite production, MHC class I expression, IFN-G production, and reduce MAP viability (46). Peripheral γδ T cells in subclinical cows have higher IFN-G production compared to clinical animals (47). Although mucosal-associated invariant T (MAIT) cells in cattle show protective roles in *Mycobacterium bovis* (48) and other mycobacterial infections (49, 50) their role in MAP infection is unknown.

### 2.2 Host-MAP interactions: adaptive immunity

Systemic immune responses to MAP infection were initially linked to early, protective T helper 1 (Th1) responses (51) with production of IFN-G and TNF-A, with JD progression associated with dominant non-protective, Th2 responses (52). However, responses are recognized as more nuanced without clear delineation of Th1/Th2 polarization (53, 54). Sheep often show a combined antibody and IFN-G response (55), and naturally infected cattle show less pronounced Th1/Th2 polarization (56). Experimentally infected calves develop early antigen-specific IFN-G and IL-10 responses in PBMCs; IFN-G persists out to 12 months while IL-10 wanes, along with increased CD25 expression on CD4^+^, CD8^+^ and ɣδ T cells (57). Subclinically infected cows have increased levels of CD4^+^, CD8^+^, and γδ TCR^+^ cell populations (58). In experimentally infected calves, systemic CD4^+^, CD8^+^ and γδ T cells expand after restimulation with MAP antigen (59) reflecting similar increases in the mid-ileum of subclinical cows (60).

Persistent MAP infection in ileal segments is associated with elevated macrophages, DCs, CD8^+^ and γδ T cells in the lamina propria, along with antigen-specific TNF-A and IFN-G responses in intestinal leukocytes (61). Subclinically infected cows have higher levels of CD4^+^ CD25^+^ T cells in the ileum versus uninfected cows (62). The lack of protective immunity despite these Th1 responses may be attributed to IL-10 producing regulatory T cells and γδ T cells suppressing pro-inflammatory responses (63), systemic and local PD1/PDL1-mediated T cell exhaustion (64), and the unresponsiveness of peripheral CD4^+^ CD25^+^ T cells that emerge in the subclinical phase (65).

Histology of granulomatous lesions reveal immune-suppressive environments. FOXP3^+^ T cells are more abundant in focal versus diffuse lesions (66). M1 macrophages predominate in focal/multifocal lesions correlating with greater MAP control, whereas M2 dominate in diffuse lesions associated with greater MAP load and high expression of IL-10 and TGF-β (67).

**Figure 1:**
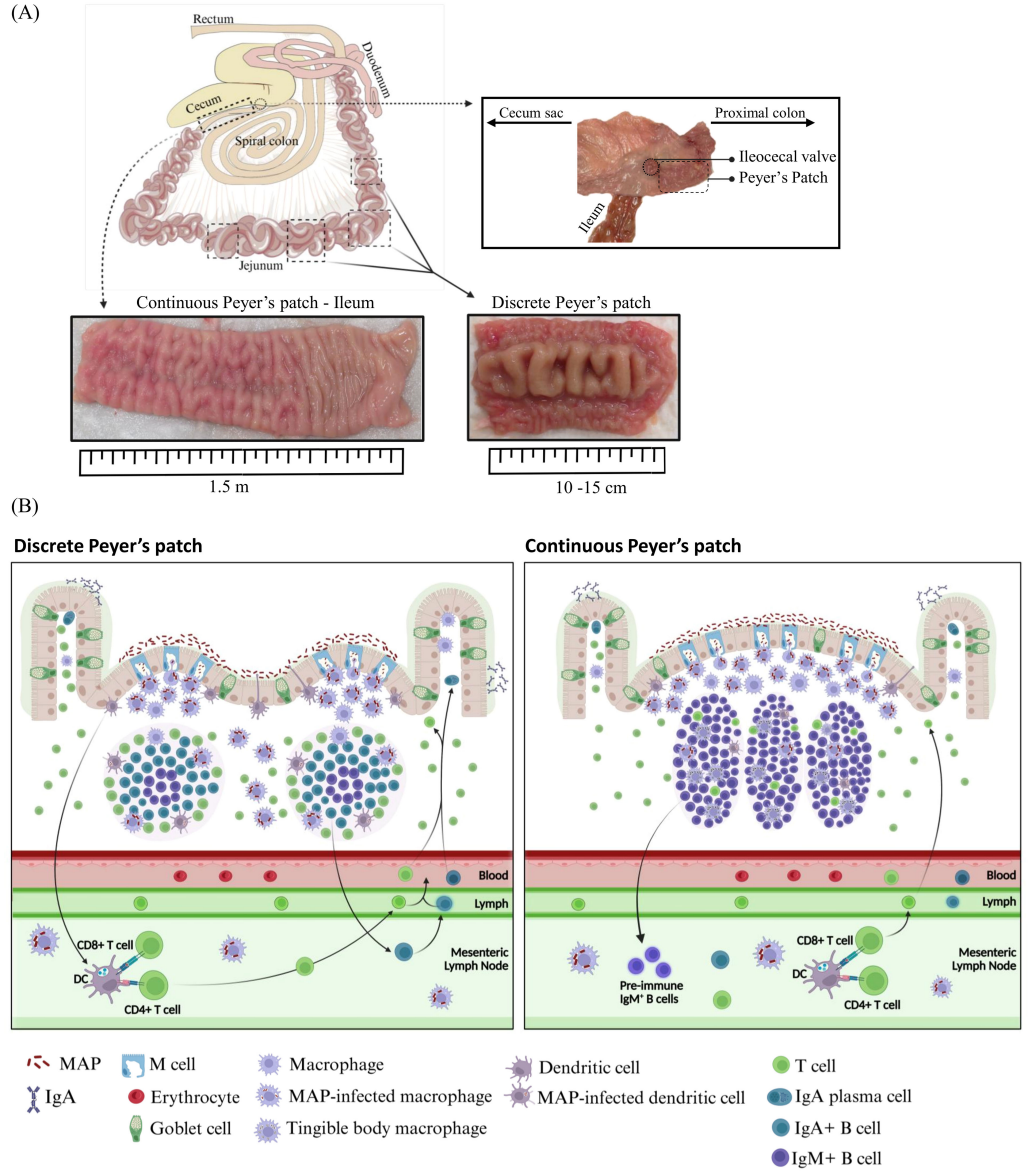
Anatomical and immunological features of bovine Peyer’s patches in the small intestine. **(A)** Schematic showing the small and large intestine in ruminants [adapted from Figure 3.22 in (71)]. Up to 40 discrete Peyer’s patches (PP), each measuring approximately 10–15 cm in length, are randomly distributed throughout the jejunum. A single continuous band of PP approximately 1.5 m in length occupies the terminal jejunum and extends throughout the ileum. A single PP is located adjacent to the ileocecal valve in the cecum; PPs have been reported in the spiral colon and less frequently in the distal rectum (72). Representative photographs are shown for the continuous, discrete, and cecal PP. **(B)** Schematic of the cellular organization and immune interactions within discrete (left panel) and continuous (right panel) PP. Discrete PPs are major mucosal immune inductions sites (73). Their lymphoid follicles contain up to 35-45% surface IgM^+^ B cells, 10-15% CD4^+^ T cells, and up to 35-50% isotype switched B cells. In contrast, the continuous PP is a primary lymphoid tissue which is the site of B cell development and generation of the pre-immune Ig repertoire (73). It has densely packed lymphoid follicles enriched in surface IgM^+^ B cells (~ 95%), less than 0.5% CD4^+^ T cells, and less than 3% isotype switched B cells. Most developing B cells undergo apoptosis within the follicles and are cleared by tingible-body macrophages, with fewer than 5% surviving and emigrating out to supply B cells to the periphery (74). The continuous PP involutes around the time of sexual maturity, whereas discrete PPs persist throughout life. In both types of PPs, follicle-associated epithelium overlying PPs contain M cells that mediate uptake and transport of material from the lumen. Just beneath this, the subepithelial dome regions consists of a palisade of mononuclear phagocytes that capture antigens delivered by M cells. In ruminants, the mononuclear phagocyte system within PPs remains poorly characterized; however, in humans and mice single-cell resolution approaches, spatial mapping, and functional studies have revealed distinct phenotypes and their unique roles in mucosal immunity (75). MAP-infected macrophages have been identified in the lamina propria, interfollicular regions, and within lymphoid follicles. Select images were generated using BioRender.com.

MAP primarily targets the ileum and ileocecal valve PP (68), though discrete jejunal PPs (69) and large intestine lymphoid tissue are also affected (70). Ruminants have two PP types in the small intestine with distinct roles in mucosal immunity (**Figure 1**) (72, 76). Discrete PPs throughout the jejunum serve as mucosal immune induction sites, while a continuous PP in the ileum is a primary lymphoid tissue that supports B cell development (74) and involutes at sexual maturity (72). MAP infection differentially affects these sites. In goats, discrete PPs and the ileocecal valve are most affected (77, 78), whereas in cattle lesions are predominate in the ileum (79).

Continuous and discrete PP are equally important sites for MAP infection. Despite a similar level of MAP tissue burden, IgA responses occur only in discrete PPs (69). Long-term, discrete PPs show reduced MAP burden, global transcriptional changes, and antigen-specific recall responses in intestinal leukocytes, whereas continuous PP maintain MAP persistence without local immune activation (80). These mucosal responses are not reflected systemically (81) highlighting the importance of local immunity for understanding MAP evasion and region-specific immunity.

## 3 Current status of MAP vaccines

### 3.1 Whole cell vaccines for MAP control

Historically, JD vaccination relied on bacterin-based vaccines such as Mycopar^®^, Silirum^®^ and Gudair^®^. Whole-cell vaccines can interfere with bovine tuberculosis (bTB) (82) and JD surveillance (83), they do not provide sterilizing immunity or DIVA (Differentiate Infected from Vaccinated Animals) compatibility, limiting their usefulness. Live attenuated vaccines (LAV), developed via deletion of virulence genes, reduce pathogenicity while preserving immunogenicity and key anti-mycobacterial immune responses such as CD8^+^ cytotoxic T lymphocytes (CTL). Parenteral vaccination with LAV ΔMAP K10/*pgsN* reduced fecal bacterial shedding in goats (84) and calves (85) and reduced granulomatous lesions in calves. Vaccine immune protection was associated with antigen-specific *IFNG, IL1A, IL17* and *TNFA* recall responses in peripheral blood mononuclear cells (PBMC) (85). Oral vaccination of calves with LAV ΔMAP A1-157/*BacA* lowered MAP burden in ileum and ileocecal valve but not jejunum (86) and increased antigen-specific IFN-G– and TNF-A–producing CD4^+^ and CD8^+^ T cells along with MAP-specific *IL12* and *IL17* recall responses in PBMCs. Gut immune protection was associated with increased CD4^+^IFNG^+^/TNFA^+^ T cells in continuous and discrete PPs, with reduced CD4^+^FOXP3^+^ cells and enhanced antigen-specific *IFNG, IL2, IL15, IL17, IP10* and *TNFA* responses in the continuous PP (87). Silirum^®^ vaccination in calves similarly revealed regional differences in vaccine-induced immunity (81). Reduced MAP burden was evident in continuous PP, but not discrete PP, and was associated with increased CD335^+^ innate lymphoid cells and local antigen-specific cytokine responses (*IL1A, IL1B, IL2B, IL21, IL27*, and *TNFA*). These studies highlight marked systemic versus mucosal and regional immune differences, which are largely overlooked in many studies. Mucosal immune responses may help to better define protective correlates based on local responses.

### 3.2 Subunit vaccine approaches for MAP control

Subunit vaccines are DIVA-compatible and can eliminate interference with bTB testing. Subcutaneous vaccination of young calves with antigen 85 complex (Ag85A-C) plus superoxide dismutase (SOD) reduced MAP bacterial burden in multiple LNs and intestinal tissue at 8 weeks post-challenge (88). Vaccine immune protection involved antigen-specific lymphoproliferation, increased CD3^+^, CD8β^+^, CD25^+^, and γδ^+^ T cells, and upregulation of *IFNG*, *IL2, IL12* and *TNFA* in antigen-stimulated PBMCs. Vaccination of one-month-old calves with a protein cocktail of four MAP antigens (MAP_1087, MAP_1204, MAP_1272c, MAP_2077c) reduced MAP burden in intestinal tissues and mesenteric LNs at 12 months post-challenge (89), and enhanced antigen-specific CD4^+^ T cells and B cells in blood and IFN-G, IL-12, and IL-10 production. Viral vector vaccines expressing MAP antigens including MAP_1589c (AhpC), MAP_1234 (Gsd), MAP_2444c (p12), and MAP_1235 (Mpa) (90) in human Adenovirus 5 and Modified Vaccinia virus Ankara reduced fecal shedding and MAP tissue burden in calves (91) and was associated with increased antigen-specific IFN-G release in whole blood and *IL22* recall response in PBMCs. Bovine herpesvirus-4 (BoHV-4) expressing modified MAP major membrane protein induced CTL responses capable of killing MAP-infected cells *ex vivo* (92). Protein-particle–based and nanoparticle platforms in mouse models reduced MAP tissue burden, induced IFN-G^+^ and TNF-A^+^ secreting CD4^+^ and CD8^+^ T cells, and generated IgA^+^ B cells in the small intestine (93, 94).

These studies demonstrate that vaccination can partially overcome MAP-induced immunosuppression observed in naturally infected animals. Vaccine-challenge models provide a framework to investigate mechanisms associated with protective immunity, which are not accessible in natural or experimental MAP infection alone but require the context of vaccine-mediated protection.

## 4 Research gaps and barriers to developing a JD vaccine

### 4.1 Antigen discovery and defining protective correlates

The MAP genome encodes 4,350 open reading frames; but only a small fraction has been explored as vaccine candidates in cattle (95). A major challenge is identifying conserved, immunodominant antigens that elicit protective immunity without interfering with bTB testing. Bioinformatics can predict potential epitopes but these require experimental validation (96, 97). Antigen discovery has largely relied on screening antigens using convalescent sera (98, 99) or measuring cell-mediated responses in blood leukocytes (100, 101). Selecting antigens reactive during early (99) or subclinical infection may represent candidates capable of blocking or controlling infection (102, 103). Antigens that elicit Th1-mediated IFN-G, CTL and cytokine responses such as *IL1A, IL1B, IL12B, IL17, TNFA* (85, 104) have been associated with protection after vaccination, but clear correlates of protection that reliably predict vaccine efficacy are lacking. Translating immune parameters (i.e., magnitude, quality, tissue localization) into meaningful outcomes such as blocking transmission, mitigating disease-associated losses in milk yield and body condition and preventing disease progression remains a challenge. Future antigen discovery should prioritize the use of intestinal leukocytes from partially protected animals rather than blood from convalescent animals, as mucosal immune responses are not reflected systemically (81) indicating that critical immune responses are being missed. Mucosal immune responses associated with protection offer one approach to select the best vaccine antigens and will also serve to guide vaccine formulation to achieve durable immunity.

### 4.2 Challenges in targeting intestinal immunity

**Figure 2:**
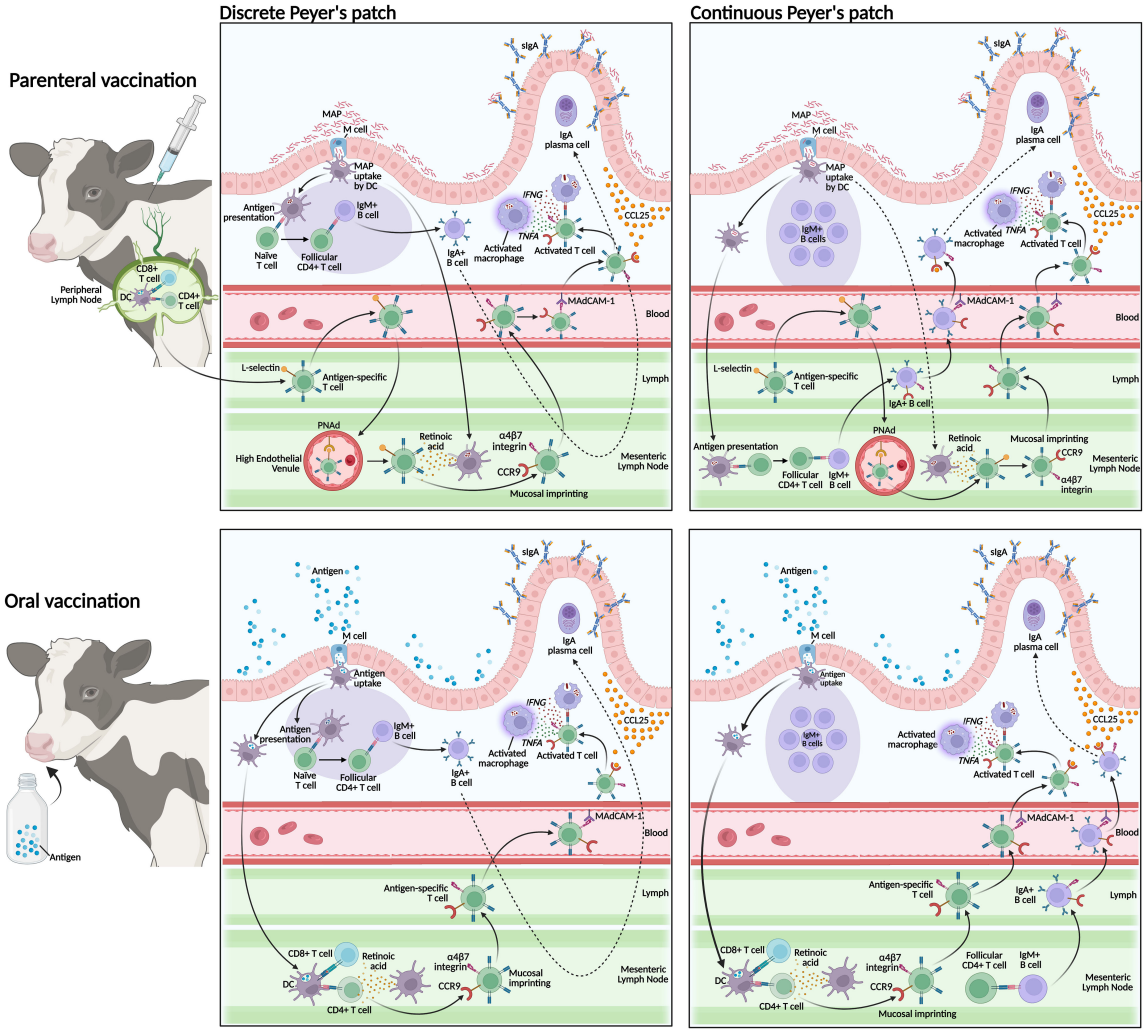
Proposed model of parenteral and oral vaccine-induced mucosal immunity in the distinct Peyer’s patches of the calf small intestine. Parenteral vaccination (upper panels) induces antigen-specific lymphocytes in peripheral lymph nodes (LN) which express homing receptors, such as CD62L (L-selectin), that facilitate trafficking into lymphoid tissues (110). Bovine Peyer’s patches express low levels of peripheral lymph node addressin (PNAd) in their high endothelial venules (HEV) which limits recruitment of these lymphocytes (111). Thus, vaccine antigen-specific lymphocytes likely seed the small intestine in low numbers prior to MAP exposure. The interaction between circulating vaccine antigen-specific lymphocytes and mucosal dendritic cells (DC) presenting MAP antigen in mesenteric LNs may be one mechanism by which these lymphocytes acquire gut-homing properties (e.g., α4β7, CCR9). This process facilitates their selective recruitment to Peyer’s patch through HEVs and the small intestine lamina propria via α4β7-MadCam-1 and CCR9-CCL25 interactions (111). Immunity induced by parenteral vaccination may rely on MAP exposure and its presentation by mucosal DCs in the gut before vaccine-activated lymphocytes can home to and populate the small intestine in sufficient numbers to confer protection. In discrete Peyer’s patches of the jejunum, naïve lymphocytes are likely activated by DCs that capture antigen in the subepithelial dome region - these cells are among the first to encounter MAP (or orally delivered vaccine antigen; lower panels) as it traverses the epithelial barrier via M cells. These mucosal DCs migrate to Peyer’s patches and mesenteric LNs generating antigen-specific T and B cell responses. The secretion of retinoic acid by mucosal DCs induces gut-homing receptors, such as α4β7 and CCR9, on lymphocytes. These imprinted cells enter circulation and are selectively recruited to the intestinal lamina propria and Peyer’s patch through α4β7-MadCam-1 and CCR9-CCL25 signaling pathways. In contrast, the continuous Peyer’s patch is unable to generate local antigen-specific responses (73, 112). We propose that immune induction instead depends entirely on antigen-bearing DCs migrating from the subepithelial dome region to mesenteric LNs, where they initiate protective T and B cell responses and imprint mucosal homing properties that supports homing back to the intestine. The extent to which Th1 responses are generated in Peyer’s patches versus mesenteric LN, and the specific subepithelial mononuclear phagocytes responsible for driving these responses remains unclear. Furthermore, the precise intestinal compartments in which Th1 cells must reside – such as the lamina propria, Peyer’s patch lymphoid follicles, or interfollicular regions – to effectively clear mucosal infection is not well defined. Although mononuclear phagocytes have been characterized in the bovine mesenteric LN there remains a lack of functional characterization, as well as detailed spatial mapping, within Peyer’s patches. DC, dendritic cell; HEV, high endothelial venule; PNAd, peripheral node addressin; MAdCAM-1, mucosal addressin cell adhesion molecule-1; Ig, immunoglobulin; CCL25, Chemokine (C-C motif) ligand 25. Image generated using BioRender.com.

Oral vaccination simulates natural MAP exposure and offers a non-invasive delivery route (86, 105). In calves, delivery in milk can bypass the forestomach via the reticular groove though older animals may require rumen-escape strategies. Key challenges include retaining antigen integrity and immunogenicity during transit, penetrating mucus and epithelial barriers ─ often via uptake by M cells ─ and stimulating subepithelial antigen-presenting cells (APC) to induce protective responses (106). While rodent studies offer solutions, bovine gut-associated lymphoid tissue architecture and PP distribution requires species-specific approaches. Oral delivery can promote gut-homing lymphocytes supporting local immunity. LAVs can elicit protective mucosal responses but may interfere with bTB testing. For subunit vaccines, safe, potent adjuvants for ruminants are needed that can bypass tolerance mechanisms to promote protective Th1-mediated responses while balancing regulatory responses to minimize excessive inflammation that risks tissue damage (107). Parenteral vaccination remains promising but shows regional variability in mucosal protection with effects in the ileum (continuous PP) but not jejunum (discrete PPs) (81). Although parenteral MAP-bacterin vaccines are effective in sheep (108) it is unclear whether peripherally activated T cells can home directly to the bovine gut as in mouse (109) or require local imprinting in mesenteric LN. Understanding these mechanisms is essential for vaccine design and adjuvant selection to induce protective intestinal immunity (**Figure 2**).

### 4.3 Tissue-resident phagocytes in MAP infection and intestinal immunity

Over the past decade single-cell RNA-sequencing and multi-color flow cytometry have revealed remarkable heterogeneity among bovine mononuclear phagocytes in blood (113), lymph nodes (114), tissues (115) and distinct regions of the small intestine (116) including sites relevant to MAP infection. However, their precise spatial distribution within bovine PPs particularly subepithelial dome regions, lymphoid follicles and interfollicular regions is not well defined. Studies in mice and human reveal region- and compartment-specific localization (75, 117). Furthermore, it remains unclear which mucosal phagocytes are susceptible or resistant to MAP and to what extent host-pathogen interactions in blood-derived macrophages and DCs reflect the biology of tissue-resident populations. Given the functional differences of ruminant PPs, this represents a fundamental knowledge gap. Addressing this is essential for identifying APCs that drive protective immune responses, including those capable of cross-presentation to generate mucosal cytotoxic T cells (118). Additionally, epithelial and goblet cells can transport antigen across the epithelium and activate underlying APCs pointing to an important but unexplored role in mucosal immunity (116, 119).

### 4.4 Regional differences in MAP infection and immunity

MAP infects multiple regions of the ruminant GI tract including jejunal and ileal PPs, mesenteric LNs, and large intestinal PPs (120–122) though the latter are understudied. Ambiguity often arises around infection of the ICV, which lacks lymphoid tissue. Infection likely involves adjacent ileal mucosa (or continuous PP in calves) or the cecal PP. More precise reporting is needed to improve interpretations across studies. Immune mechanisms controlling MAP at these distinct immunological sites remain poorly defined, with evidence of region-specific responses in discrete versus continuous PP in calves (81) and associated mesenteric LNs in cows (122). Reliance on blood-based immune responses limits our view of host-MAP interactions due to differences observed with mucosal immune responses. This is particularly relevant given the temporal changes associated with continuous PP which involute after sexual maturity (3). In calves, MAP localizes to submucosal and interfollicular regions (81, 86) whereas in adult cattle with clinical JD it is predominantly in the lamina propria macrophages of the ileum (123). How MAP or MAP-infected cells traffic between these compartments over time underscores the need for long-term, region-specific studies to better understand pathogenesis.

### 4.5 The role of B cells, antibodies, and passive immunity in MAP control

A key challenge is determining the optimal age for vaccination as calves may be infected with MAP at or shortly after birth (124). Early life immunity is essential, but vaccination is typically delayed weeks to months after birth to avoid maternal antibody interference creating a window of high susceptibility during which susceptibility to MAP is greatest (125). Enhancing maternal immunity, specifically transfer of MAP-specific antibodies, may help control infection in neonatal calves. *In vitro*, IgG1 antibody-mediated opsonization can modulate macrophage responses limiting the ability of MAP to impair apoptotic pathways (126, 127). Preincubation of MAP with systemic antibodies from convalescent cows limited bacterial invasion and attenuated initial histological changes in the mucosa using a calf ileal loop model (128). However, in naturally infected cows maternal anti-MAP antibodies in colostrum had minimal impact on whether the daughters became MAP shedders (129). Discrepancies between these observations may reflect variations in antibody isotypes and/or repertoires which were not characterized. Vaccine-induced antibodies may yield different outcomes than those from natural infection.

Growing evidence supports a protective role for B cells in tuberculosis mediated by antibodies, cytokine secretion, antigen presentation, and interactions with other immune cells (130). In sheep, vaccine-mediated protection to MAP has been linked to increased intestinal B cell responses (131) and gene expression associated with humoral immunity (132). Despite the historical associations between B cells/antibodies and disease progression (133) a mucosal-focused perspective involving B cells and intestinal IgA and IgG may reveal protective roles. Together, these findings conceptually support that MAP-specific B cells and antibodies may contribute to protective immunity, however important knowledge gaps remain.

## 5 Conclusion

A major barrier to advancing an effective JD vaccine rests in the incomplete understanding of mucosal immune responses both at the site of infection and in the face of neonatal immunity. The GI tract is a highly specialized immune environment with regionally distinct areas, yet the mechanisms governing protective versus permissive responses to MAP are poorly defined. This gap limits the ability to identify relevant vaccine antigens, design delivery systems to stimulate intestinal immunity, and elicit durable, protective immunity where it is most needed. Addressing these research gaps can enable rational antigen discovery, help define correlates of protection, and ultimately improve vaccine approaches. A comprehensive understanding of the ruminant mucosal immune system will not only serve to advance vaccine development for JD but will also broaden our insights into host-MAP interactions.
